# Prognostic associations of cortical gyrification in minimally medicated schizophrenia in an early intervention setting

**DOI:** 10.1038/s41537-022-00296-y

**Published:** 2022-10-29

**Authors:** Pan Yunzhi, Xudong Chen, Eric Chen, Edwin Lee, Liu Zhening, Xuan Ouyang, Lena Palaniyappan

**Affiliations:** 1 Institute of Mental Health, Second Xiangya Hospital, Central South University, Changsha, PR China; 2 Robarts Research Institution, University of Western Ontario, London, ON Canada; 3 Queen Mary Hospital, University of Hong Kong, Hongkong, PR China; 4 Department of Psychiatry, University of Western Ontario, London, ON Canada; 5 Douglas Mental Health University Institute, Department of Psychiatry, McGill University, Montreal, QC Canada

**Keywords:** Schizophrenia, Biomarkers

## Abstract

The aberration in cortical gyrification seen in schizophrenia likely originates in the earliest phase of life, as gyrification begins in utero and reaches its peak in infancy. However, emerging observations have indicated a later reduction in gyrification, especially in early adulthood, may also occur in schizophrenia. At present, it is unclear whether the baseline and later gyrification reduction has any prognostic importance in schizophrenia. We address this question in a longitudinal design in patients minimally medicated at inception. About 108 minimally medicated (duration of medication = <14 days of antipsychotics) patients and 106 healthy controls underwent structural magnetic resonance imaging, with 34 patients being selectively re-scanned when clinically stable following antipsychotic treatment. The cortical surface from each structural image was reconstructed, and the local gyrification index and cortical thickness were computed for each vertex on the surface. We found minimally medicated schizophrenia patients during the first episode had a relatively higher gyrification in bilateral supramarginal, left superior temporal, and right posterior cingulate and paracentral regions. However, poor prognostic features were more likely in patients with lower baseline gyrification. Longitudinal reductions in left superior parietal and right precentral gyrification were associated with lower improvements in both positive and negative symptoms over time. The spatial pattern of longitudinal changes in gyrification was distinct from the changes in cortical thickness. These results indicated that schizophrenia is characterized by a relative hypergyrification in parieto-temporal and medial cortical areas at a group level at first presentation, but poor outcomes relate to lower-gyrification elsewhere both at the onset and during the early course. The early post-onset reduction of gyrification is rather limited in space and magnitude, but occurs unrelated to the progressive thinning, representing a distinct, prognostically important structural trajectory.

## Introduction

Schizophrenia is often considered to be a neurodevelopmental illness^1,2^ with progressive changes in cortical gray matter occurring in the initial years of its symptomatic onset. The study of cortical surface anatomy, especially the folding pattern or gyrification^3–5^ and thickness of the cortex provides insights into the maturational pathophysiology of schizophrenia^6^. Of note, several prior studies have demonstrated an association between poor prognostic features and reduced gyrification in psychosis (reduced response to antipsychotics^7^, emergence of treatment resistance^8^, residual symptom burden^9^).

Gyrification patterns are established predominantly in the late second to third trimesters of pregnancy and remain relatively stable after postnatal week;^6,10,11^ aberrant gyrification in adults with schizophrenia is often interpreted as a sign of an in utero pathology^12^. This interpretation is supported by studying obstetric complications; preterm-born adults show widespread reductions in gyrification similar to the pattern seen in patients with established schizophrenia^13^. Further, genetic loading for schizophrenia relates to reduced gyrification; this has been demonstrated using multiple methods of inquiry now^14–17^. This has led to the inference that a developmental insult in utero leads to reduced gyrification that affects the prognosis of schizophrenia^18^. However, several lines of inconsistent evidence also exist.

Firstly, many studies have failed to observe a schizophrenia-related diagnostic effect on gyrification^19^ (see ref. ^20^ for a review), while some show increased gyrification^21–23^ when compared with healthy controls (HCs). An important confound in morphometric studies of schizophrenia is the long-term exposure to antipsychotics^24^. A literature review of prior vertex-by-vertex whole-brain comparisons indicates that most published studies (*n* = 16) have recruited patients that received antipsychotics for many years (>3 years; See Supplementary Table 1). Even first-episode samples predominantly include patients who have had several weeks of exposure to antipsychotics^25–28^. While the extent to which gyrification is affected by antipsychotics is still unknown, studying minimally medicated patients in an early intervention setting assumes crucial importance to disentangle illness-related effects from the potential confounds introduced by variations in dose/duration of medication use.

Secondly, longitudinal studies indicate that gyrification patterns, measured using the commonly employed “buried cortex” approaches^29^ among several well-established methods^29,30^, are not always invariant in adult life; they change over time, at least in the early stages of schizophrenia^24,27,31^. It is not clear if this later change reflects a progressive cortical atrophy process in schizophrenia, in which case, we can expect a longitudinal reduction in cortical thickness to accompany the reduction in gyrification over time. This scenario, if present, will support a recent proposal from Sasabayschi and colleagues^32^: secondary changes in gyrification occur as a result of the “mechanical stress of progressive tissue reduction”. Examining concurrent changes in thickness and gyrification over time is important to answer this question. Previous studies also found the heterogeneity of cortical gyrification across subgroups of schizophrenia patients^15,33,34^. Given our previous work found different abnormal patterns of cortical thickness in schizophrenia^35^, we will also verify whether gyrification has similar patterns.

Another critical issue in the study of gyrification is the use of multiple morphometric methods that rely on various physical aspects of the folding pattern: some on sulcal depth, width, and curvature, while others on the ratio of buried vs. unburied cortices (such as the most used method—LGI). In addition to the effect of medication, variations in measurement accuracy may also affect the direction and magnitude of the reported results to date (see Supplement Table 1); these issues are critical when considering a longitudinal study.

In the present study, we focus on clarifying the prognostic associations of gyrification in the early stages of schizophrenia. To this end, we first evaluate the gyrification index in a vertex-wise fashion across the entire brain in minimally medicated (average 6.4 days of lifetime exposure) patients and study the relationship between gyrification and core clinical symptoms and duration of untreated illness at baseline. We then quantify the degree of progressive changes in gyrification by following up a sub-sample of patients after initiating treatment, at a time of clinical stability (i.e., when not hospitalized anymore) and relate this to the degree of clinical response. We scrutinize the relationship between thickness and gyrification and test the mechanical stress hypothesis by (1) identifying a subgroup of patients with reduced thickness at baseline and studying if they exhibit concomitant gyrification deficits (2) studying the patterns of associations between longitudinal changes in thickness and gyrification. Given the emerging observations of relative “hypergyria” from early stage samples^36–40^, we expected patients to show a diffuse increase in gyrification at the outset, in relation to symptom burden and illness duration at the outset. Given the inconsistency in existing literature, we did not predict specific regional changes a priori and use two-tailed tests for all analysis.

## Methods

### Participants

In this study, we collected cross-sectional and longitudinal data. At baseline, 108 minimally medicated (≤14 days) patients with schizophrenia and 106 age- and sex-matched healthy controls (HC) were included. Patients were recruited from inpatient and outpatient clinics of the Institution of Mental Health in Second Xiangya Hospital and Queen Mary Hospital of the University of Hong Kong and met DSM-IV criteria for schizophrenia. The diagnosis was made by a qualified psychiatrist through the SCID checklist for schizophrenia in the hospital setting. This diagnosis was later rechecked after 6 months through a face-to-face interview (again in the clinical setting) or via telephone contact and/or review of hospital records when this was not possible. Only participants with a stable diagnosis of DSM-IV schizophrenia at 6 months are included in the study.

All patients had ≤14 days of lifetime antipsychotic exposure, were right-handed (assessed by the Annett Hand Preference Questionnaire^41^) and completed ≥9 years of formal schooling. Healthy controls (HC) were recruited from the community, hospital staff and schools. The exclusion criteria included: (1) meet the diagnosis of any mental disorder except schizophrenia in DSM-IV; (2) any reported history of substance-related disorders, neurological disorder, or serious physical illness in themselves or their first-degree relatives; (3) any contraindication for MRI; (4) left-handedness (as China has a usually low prevalence of left-handedness, exclusion was more practical than case-control matching)^42^; (5) history of brain injury or conscious coma; (6) intellectual disability (IQ < 70); and (7) previous electroconvulsive therapy. Patients were followed up after at least one month of antipsychotic treatment (follow-up time: between 1 and 11 months, mean = 3.62 months, SD = 2.58). Thirty-four patients completed follow-up symptom measurements and the required MRI examination. Demographic information is shown in Table 1. The reasons why the patient was not followed up successfully include: (a) Refuse to continue to participate in the project; (b) Electroconvulsive therapy during follow-up; (c) Unable to contact.

**Table. 1:** Demographic features of the participants.

	Baseline			Follow up
	Patients (*n* = 108)	Controls (*n* = 106)	*p*	Patients (*n* = 34)
Center (CS/HK)	86/22	79/27	0.376	16/18
Sex(M/F)	62/46	58/48	0.692	14/20
Age (year)	24.97(7.89)	23.67(5.12)	0.153	28.12(10.60)
Onset age	23.83(8.05)			27.64(10.93)
Duration of illness (month)	15.13(22.75)			7.45(11.07)
Daily dosage of Medication (CPZ/day)	220(189)			226(197)
Duration of medication exposure (days)	6.4(4.4)			115.49(78.45)
Follow-up duration (month)	—			3.62(2.58)
Total score of symptom	51.94(31.39)			16.67(14.67)
SAPS score	23.37(14.78)			1.73(3.49)
SANS score	28.57(24.69)			14.88(14.29)
N-back (accuracy rate)	0.55(0.22)	0.71(0.21)	0.000**	
Estimated total intracranial volume	1.48E + 6(2.38E + 5)	1.31E + 6(2.39E + 5)	0.000**	

N-back task was just assessed in the dataset from Changsha, and we only use the data from this center when we do the analysis related to N-back task.

### Clinical and cognitive assessment

At baseline measurement, psychopathology was assessed by a qualified psychiatrist in the research team using the Scale for the Assessment of Positive Symptoms (SAPS) and the Scale for the Assessment of Negative Symptoms (SANS). The N-back^43^ task (including 0-back and 2-back) was used to assess working memory^44,45^. Given our prior finding linking gyrification and n-back-related brain connectivity^46^, we performed a parametric N-back task for participants on an E-prime hardware system for 8 min and 16 s. All stimuli were sequences of white capital letters on a black background, presented centrally (500 ms duration, 1500 ms inter-stimulus interval) in a pseudo-random order. The task performance, as represented by the accuracy of responses, was calculated for each participant.

At follow-up measurement, SAPS and SANS were used to assess psychopathology. Efficacy (evaluated by the change of the sum of SAPS and SANS) and change of positive or negative symptoms were calculated using the following formula: [Baseline – Follow up]/Baseline. During the follow-up period, patients were receiving stable antipsychotic treatment (no change in dose or medication type for at least the last 4 weeks before follow-up assessment) (Table 1). This was determined from self-reports of patients and confirmation from clinicians and carers (accompanying family members where appropriate). Clinicians indicated the timing for follow-up scans based on the first instance of reported clinical stability. This was defined as the earliest follow-up phase when patients reached a state of stable remission (total score reduction rate > 30% or all items scores on SAPS and SANS ≤ 2^47^) with no medication changes in the preceding month for reasons of symptom exacerbation. While 40 subjects were referred for follow-up based on clinical impression, six did not satisfy this stability criterion when symptom scores were evaluated, and the duration of stability and treatment was considered. Thus, 34 patients that reached stable remission levels entered the follow-up analysis. To compare the dose between groups, we converted different drugs into chlorpromazine equivalence according to Leucht and colleagues’ methods^48,49^. The authors assert that all procedures contributing to this work comply with the ethical standards of the relevant national and institutional committees on human experimentation and with the Helsinki Declaration of 1975, as revised in 2008. And all participants gave written informed consent to the study approved by the local Ethics Committee of Second Xiangya Hospital and Queen Mary Hospital.

### Magnetic resonance image (MRI) acquisition

The participants were scanned using a Philips 3.0 Tesla MRI scanner. At Changsha center, T1-weighted magnetic resonance imaging data were acquired using a three-dimensional spoiled gradient-echo (SPGR) pulse sequence from the sagittal plane, scanning parameter as follow: TR = 7.5 ms, TE = 3.7 ms, flip angle = 8°, 180 slices, the field of view (FOV) = 240 mm^2^ × 240 mm^2^, and slices were contiguous with a slice thickness of 1 mm. At Hong Kong center, the MRI parameters of the 3D magnetization-prepared rapid gradient-echo (MPRAGE) sequence were as follows: TR = 7.0 ms, TE = 3.2 ms, flip angle = 8°, number of slices = 160, FOV = 240 mm^2^ × 240 mm^2^, slice thickness = 1 mm. Then, 7 min 12 s long echo-planar functional images were acquired immediately after the T1 scans, with no other sequence in between. At both sites, resting fMRI acquisitions were used to estimate head motion by applying the rigid-body transformation, and any subject who moved >2 mm was not included for further analysis. Thirteen subjects were excluded in total for this reason.

### Image processing

The cortical reconstruction and preprocessing of T1-weighted images were carried out using FreeSurfer (http://surfer.nmr.harvard.edu, version 5.3.0). This method followed a standard auto-reconstruction algorithm, including non-uniform intensity normalization, removal of non-brain tissue, affine registration to the Montreal Neurological Institute (MNI) space and Talairach transformation, and segmentation of gray/white matter tissue. The technical details of the processing are described in previous publications^50,51^. The reconstructed images and the segmentation of gray-white matter were inspected visually by an experienced researcher, and movement-related quality checks were made as per prior criteria^4^. Any inaccuracies in registration or tissue segmentation were modified according to the tutorial (http://surfer.nmr.mgh.harvard.edu/fswiki/Tutorials). After preprocessing, we obtained cortical thickness values for each vertex and the eTIV (estimated Total Intracranial Volume) for the whole brain.

The vertex-wise method (advocated by Schaer et al.^52^) was used to continuously assess the local gyrification index (LGI) of the entire cortex. This method is an extension of classical two-dimensional GI measurement that calculates the ratio of the pial perimeter over the outer perimeter on coronal sections^53^. It provides an LGI for each vertex on the cortical surface, which reflects the amount of cortex buried in its immediate locality.

### Statistical analysis

#### Cross-sectional comparison

The differences in demographic characteristics and clinic data between groups were compared using the independent two-sample *t*-test, analysis of variance (ANOVA), or nonparametric tests as appropriate by SPSS19.0. Each vertex-wise LGI value was mapped on a common spherical coordinate system (fsaverage) to enable group comparisons. Query-Design-Estimate-Contrast tool (QDEC) in the Freesurfer was used to generate the contrasts. Then the smoothing calculation was conducted using a 5-mm Gaussian kernel^15,23^. A general linear model (Glm) controlling for the effect of age, center, and sex was used to compute differences in gyrification between the groups at each vertex of the right and left hemispheric surfaces. Cohen’s d was calculated to evaluate the effect size of group-level comparisons. To correct for multiple testing, we used Monte-Carlo simulations (*n* = 10,000) and identified clusters that survived a type-1 error rate of 5% at a cluster inclusion threshold of *P* = 0.001.

The onset is calculated from the date with noticeable psychotic symptoms (delusions, hallucinations, or disorganized speech/behavior) reported by the patient himself and his family, and the duration of illness was determined on the basis of the onset date. The correlation analysis between LGI and clinical features (Duration of Illness (DoI), SAPS total score, SANS total score, and N-back) was conducted in patients using the general linear model interface of QDEC with age, center, and sex as covariates. LGI at baseline was studied in relation to treatment response (decreased rate of total, positive, and negative symptoms). We again used Monte-Carlo simulations (*n* = 10,000) to identified clusters that survived a type-1 error rate of 5% but at a lower cluster inclusion threshold of *P* = 0.01, as the clinical variables of interest were not fully independent variables (|r| = 0.0001 to 0.2245). Note that most correlations also survived a more stringent threshold of 0.001, except for antipsychotic dose relationships.

#### Longitudinal comparison

A vertex-wise longitudinal imaging preprocessing^54^ was conducted using Freesurfer as described in https://surfer.nmr.mgh.harvard.edu/fswiki/FsTutorial/LongitudinalTutorial. Specifically, an unbiased within-subject template space and image^55^ is created using robust, inverse consistent registration. Several processing steps, such as skull stripping, Talairach transforms, atlas registration, as well as spherical surface maps and parcellations, are then initialized with common information from the within-subject template, significantly increasing reliability and statistical power^54^. After preprocessing, the changes in LGI and thickness were calculated as [Follow up − baseline]/baseline value. Freesurfer’s Query-Design-Estimate-Contrast (QDEC) interface was used for statistical analysis, with one-sample *t*-test between [Follow up – baseline]/baseline vs. null applied to estimate the significance of paired difference, adjusted for baseline effect. We also computed the summary effect sizes as per Goulet-Pelletier & Cousineau^56^ [under the assumption of homogeneity of variances, normal distribution for repeated measures design and two-group designs]. A general linear model controlling for the effect of age, center, sex, and follow-up interval was used to compute the correlation between the change of LGI and the change of clinical symptom at each vertex of the right and left hemispheric surfaces. To correct for multiple testing, we used Monte-Carlo simulations (*n* = 10,000) and identified clusters that survived a type-1 error rate of 5% at a cluster inclusion threshold of *P* = 0.01. We report the cluster-wise *p* values (CWP) after correction.

#### Cluster analysis

K-means clustering method and GAP statistics were used to identify clusters of participants who shared similar patterns of cortical morphology. K-means clustering was applied to all participants, including HCs based on the morphology of 68 brain regions (Desikan–Kiliany Atlas), separately for cortical thickness and LGI, according to our previous work^35^.

## Results

### Demographic and clinical characteristics

Demographic and clinical data of patients and healthy controls is presented in Table 1. There were no significant differences in demographic characteristics including sex (χ^2^ = 0.156, *p* = 0.692), age (*t* = 1.435, *p* = 0.153) and center (χ^2^ = 0.785, *p* = 0.376), indicating that both patients and controls had comparable probability of coming from either centers. The patients had lower accuracy of N-back (χ^2^ = 17.082, *p* = 0.000) and higher eTIV (*t* = 5.214, *p* = 0.000) compared with healthy controls. At baseline, the mean duration of illness (DoI) was 15.13 ± 22.75 months. For the longitudinal sample, the mean follow-up interval was 3.62 ± 2.58 months.

### Group differences in gyrification at baseline

**Fig. 1:**
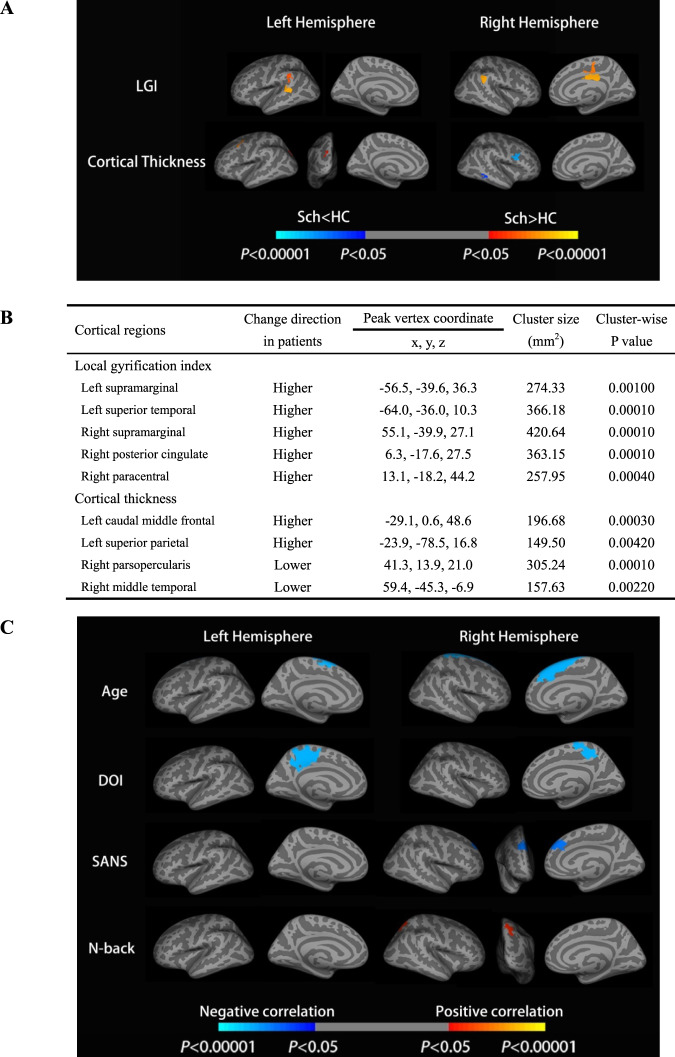
Clusters showing differences in the LGI, cortical thickness between schizophrenia patients, and healthy controls at baseline. **A**, **B** Cortical statistical maps displaying an increased LGI in schizophrenia patients compared with healthy controls. The maps are shown for the bilateral supramarginal, left superior temporal, right posterior cingulate, and right paracentral, respectively. The horizontal bar shows *p* values after correction for multiple comparisons using Monte-Carlo simulations (threshold = 0.001); the blue regions show clusters where values are lower in schizophrenia and the red regions are clusters where values are higher in schizophrenia (vs. healthy controls) with *p* values from 0.05 to 0.00001. **C** Cortical statistical maps displaying the correlation between the LGI and clinical variables in the patient group at baseline. The horizontal bar shows *p* values after correction for multiple comparisons via Monte-Carlo simulations (threshold = 0.01), where the blue region shows Sch < HC with *p* values from 0.05 to 0.00001 and the red region shows Sch > HC with *p* values from 0.05 to 0.00001. DOI duration of illness in months, SANS schedule for assessment of negative symptoms; For the N-back task, accuracy measures were employed in the analysis.

Whole-brain vertex-wised group analysis with center, sex, and age as covariates revealed two clusters in the left hemisphere and three clusters in the right hemisphere with a significant increase in LGI in patients compared with healthy controls. These clusters included the bilateral supramarginal, left superior temporal, right posterior cingulate, and right paracentral. With respect to thickness, significant cortical thinning was observed in the right parsopercularis and middle temporal regions in patients compared with healthy controls. The largest region was parspoercularis. Meanwhile, we observed increased cortical thickness in the left caudal middle frontal and superior parietal in patients compared with healthy controls. These group differences are shown in Fig. 1. Besides, a General Linear Model analysis (glm) with age and sex showed that there was no significant difference in LGI and cortical thickness between centers 1 and 2 after correction.

### Correlational analysis at baseline

At baseline, we analyzed the correlation between LGI with DoI, SAPS total score, SANS total score, and N-back in drug-naive patients (shown in Fig. 1). The correlation analysis between LGI and age at baseline showed that LGI decreased with age in bilateral superior frontal (CWP_left_ = 0.0001, CWP_right_ = 0.0001). A negative correlation between LGI and DoI was observed in the bilateral precuneus (CWP_left_ = 0.0001, CWP_right_ = 0.0001). For the clinical symptom, there was a certain region in the right superior frontal (CWP = 0.0004) that showed a negative correlation between LGI with SANS total score, but no corrected cluster correlated to SAPS. In the cognitive aspect, the higher N-back accuracy was associated with the higher LGI in the right superior parietal region (CWP = 0.0031). In the aspect of treatment response, there was no correlation between LGI at baseline and total treatment response and decreased rate of SAPS scores, but LGI at baseline was negatively correlated with a decreased rate of SANS scores (Supplementary Fig. 2).

### The relationship between longitudinal changes of gyrification and clinical changes

**Fig. 2:**
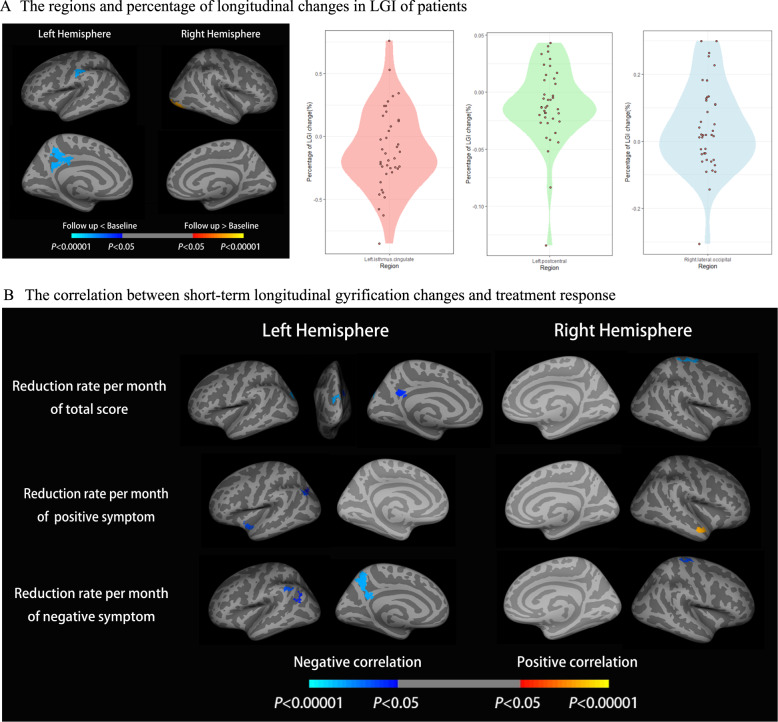
The longitudinal changes in gyrification and its negative correlation with treatment response. **A** The regions and percentage of longitudinal changes in LGI of patients; The method used in the above results is a one-sample *t*-test of [follow up - baseline]/baseline against null and paired *t*-test; in violin plot, each data point refers to the actual percentage of longitudinal change averaged across the cluster representing the labeled region from the vertex-wise analysis (i.e., a spatially contiguous cluster). The distribution across either side of null reflects the observation that some subjects had an increase while others had a decrease over time on average, with the effect sizes in Table 2 indicating a relatively minor magnitude of time-varying changes despite their statistical significance in the within-subject analysis; **B** Cortical statistical maps displaying the negative correlation between the longitudinal gyrification changes and treatment response. Clusters in cold blue indicate that a longitudinal reduction in LGI in these regions relates to less symptomatic improvement in total, positive and negative symptom scores in the sub-panels of cortical surfaces displayed from top to bottom. The horizontal bar shows *p* values after correction for multiple comparisons via Monte-Carlo simulations (threshold = 0.01), where the blue region shows Sch < HC with *p* values from 0.05 to 0.00001 and the red region shows Sch > HC with *p* values from 0.05 to 0.00001.

In the longitudinal analysis, the percentage of gyrification change was calculated using [Follow up − Baseline]/Baseline. A reduction in gyrification was observed in left isthmus cingulate (CWP = 0.0001) and left post central (CWP = 0.0001), and an increase was observed in right lateral occipital (CWP = 0.0001), but the mean change over the observed span of time in each of this region was of small magnitude (Fig. 2A; percentage changes and effect-sizes presented in Table 2 and Supplement Fig. 2). After adjusting for follow-up interval, imaging center, sex, age at baseline, we found that patients with smaller reductions in gyrification over time in left superior parietal (CWP = 0.0001), left precuneus (CWP = 0.0029), and right precentral (CWP = 0.0001) had better treatment efficacy (greater reduction in total scores of SAPS and SANS) (Fig. 2B). When the analysis was limited to improvements in positive symptoms (decrease in total SAPS score), the better treatment response (positive symptom reduction) was associated with smaller reductions of gyrification in left superior temporal (CWP = 0.0012) and left inferior parietal (CWP_Cluster1_ = 0.0016, CWP _Cluster2_ = 0.0093), but was also related to greater reduction of gyrification in right superior temporal region (CWP = 0.0001) (Fig. 2B). When limited to improvements in negative symptom, greater reduction in total SANS score was associated with smaller reductions in gyrification of left precuneus (CWP = 0.0001), left inferior parietal (CWP_Cluster1_ = 0.001, CWP _Cluster2_ = 0.0043), and right precentral (CWP = 0.0013) (Fig. 2B). After permutation testing, there was no significant difference in cortical thickness between follow-up and baseline.

**Table 2:** The effect size of cross-sectional analyses, longitudinal analysis and correlation analyses. **A**: The effect size of cross-sectional analyses. **B**: The effect size of longitudinal analysis. **C**: The effect size of correlation analysis.

	SZ	HC	*P*	Cohen’s d	[HC-SZ]/HC% change
Local gyrification index					
Left supramarginal	3.4482 ± 0.2473	3.3754 ± 0.2737	0.042	0.280	−2.16%
Left superior temporal	3.7671 ± 0.2533	3.5418 ± 0.4191	<0.001	0.650	−6.36%
Right supramarginal	3.3491 ± 0.2320	3.2623 ± 0.2275	0.006	0.379	−2.66%
Right posterior cingulate	2.2038 ± 0.2147	2.1361 ± 0.2178	0.023	0.357	−3.07%
Right paracentral	2.3175 ± 0.1928	2.2499 ± 0.1882	0.01	0.314	−3.00%
Cortical thickness					
Left caudal middle frontal	2.1208 ± 0.3169	2.0230 ± 0.2190	0.009	0.360	−4.83%
Left superior parietal	1.8151 ± 0.2016	1.7409 ± 0.1676	0.004	0.398	−4.26%
Right parsopercularis	2.3426 ± 0.2423	2.5015 ± 0.2100	<0.001	0.697	6.35%
Right_middletemporal	2.7138 ± 0.2776	2.7982 ± 0.1962	0.011	0.349	3.02%

Regions of results	Mean (baseline)	Mean (follow up)	*P* (paired *t*-test)	Cohen’s d	Baseline-FUP/Baseline %change
Left supramarginal	3.4150 ± 0.2741	3.4114 ± 0.2738	0.049	0.0131	0.104%
Left superior temporal sulcus (posterior)	3.7050 ± 0.2597	3.7046 ± 0.2597	0.037	0.0016	0.011%
Right supramarginal	3.2852 ± 0.2150	3.2865 ± 0.2151	0.047	0.0062	−0.041%

Regions	Related variables	*p* value	*r* value
The correlation between the LGI and clinical variables in a patient group at baseline			
Left superior frontal	Age	<0.001	−0.366
Right superior frontal	Age	<0.001	−0.335
Left precuneus	DoI	0.042	−0.199
Right precuneus	DoI	0.049	−0.192
Right superior frontal	SANS	<0.001	−0.362
Right superior parietal	N-back	0.009	0.325
The correlation between longitudinal changes in gyrification and treatment response			
Left superior parietal	Change in total symptom	0.007	−0.452
Left precuneus	Change in total symptom	0.031	−0.370
Right precentral	Change in total symptom	0.005	−0.473
Left superior temporal	Change of positive symptom	0.023	−0.390
left inferior parietal ROI1	Change of positive symptom	0.017	−0.406
left inferior parietal ROI2	Change of positive symptom	0.044	−0.347
Right superior temporal	Change of positive symptom	0.003	0.487
Left precuneus	Change of negative symptom	0.004	−0.482
left inferior parietal ROI3	Change of negative symptom	<0.001	−0.592
left inferior parietal ROI4	Change of negative symptom	0.005	−0.470
Right precentral	Change of negative symptom	0.012	−0.428

**Fig. 3:**
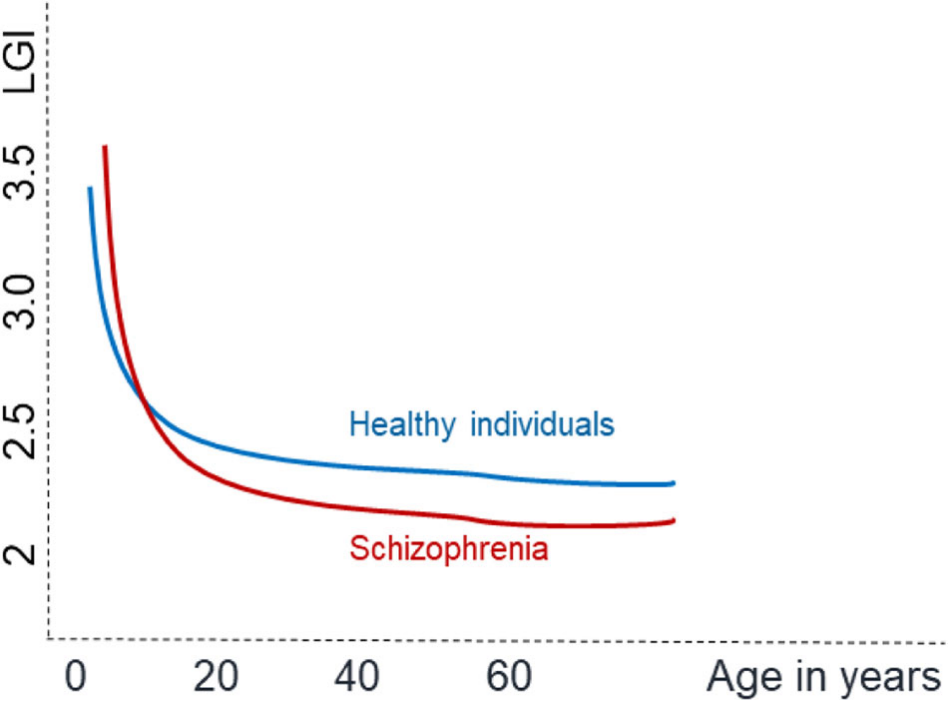
A hypothetical summary indicating the possibility of LGI peaking later but dropping more steeply during the lifespan in patients with schizophrenia compared to healthy individuals. According to this model, hypogyria may be apparent in patients studied in very early stages and in chronic, established stages. Nevertheless, a hypergyric pattern may be prominent between these two phases. Note: this hypothesis is not directly tested in the current study and regional variations in this model may exist.

Left inferior parietal ROI1 and left inferior parietal ROI2 correspond to the regions shown in the “positive symptoms” row of Fig. 3B; left inferior parietal ROI3 and left inferior parietal ROI4 correspond to the inferior parietal regions shown in the “negative symptoms” row of Fig. 3B.

### The effect of antipsychotic medication

At baseline, the minimal exposure that patients had did not show a dose-related correlation with LGI, but higher doses related to lower cortical thickness in the left superior parietal (CWP = 0.0002) even at this very early stage of treatment (Supplementary Fig. 3).

At follow-up, a higher daily dose at the time of the second scan and a higher cumulative dose of medication exposure were both related to higher LGI in the right medial orbitofrontal region (CWP = 0.0043, 0.0001) (Supplementary Fig. 4). Higher daily dosage also related to higher cortical thickness in the left caudal middle frontal cortex (CWP = 0.0069) at follow-up, while higher cumulative exposure related to higher cortical thickness in right lateral occipital (CWP = 0.0033) and superior temporal (CWP = 0.0071) (Supplementary Fig. 4).

### The uncorrelated relationship between gyrification and cortical thickness

The results above found no significant change in cortical thickness during the follow-up period, but there were regional reductions in LGI (Fig. 2). Further K-means clustering analysis at baseline showed that all patients formed a single cluster based on gyrification patterns noted at baseline. But for cortical thickness, a solution with two subgroups was found—one with notable, widespread reduction in thickness (the impoverished group *n* = 64) compared to the other, un-impoverished group (*n* = 44). When we compared LGI in a vertex-wise fashion between the two thickness-based subgroups, we found no significant differences in gyrification (even at a cluster inclusion threshold of *p* = 0.01).

## Discussion

To our knowledge, this is the first longitudinal whole-brain vertex-wise examination of gyrification initiated from a minimally medicated state of first-episode schizophrenia. Three prominent findings emerged: first, higher gyrification of small to medium effect size is prominent at first presentation with schizophrenia. In our sample, this was localized to bilateral supramarginal, left superior temporal, right posterior cingulate, and right paracentral regions. Second, poor prognostic features (longer illness duration and reduced cognitive performance) had a small to a medium-sized relationship with lower, not higher gyrification (bilateral precuneus). Treatment response is diminished in those with more notable reductions in left superior parietal and right precentral gyrification over time, though the actual reduction in LGI itself was very small during the short interval of follow-up. Third, the trajectory of cortical thickness differs from that of gyrification; the subgroup derived from statistical clustering analysis with notable thickness reduction does not show a concomitant reduction in gyrification at baseline or over time, no linear relationships with progressive thinning is notable in regions with longitudinal changes in gyrification, and the spatial distribution of progressive thinning in the longitudinal patient sample differs from that of gyrification changes.

Increased gyrification at the time of the first presentation of schizophrenia is now emerging as a consistent feature across studies where such samples have been collected^31,39,51,57,58^. This “hypergyric” pattern is also seen in other mental disorders^59,60^, and contrasts with more established cases of schizophrenia where the predominant pattern is one of reduced gyrification^4,14,61^. It is important to note that the small to medium-sized increase seen in these “hypergyric” regions do not appear to be fully ameliorated in later-stage samples in patients; they become less prominent but still detectable when the search space is restricted to smaller volumes (see refs. ^4,62^). While an accelerated decline in gyrification over a longer time span has been previously shown in patients^27,31^, the prognostic implications of this, especially during an early treatment phase, were hitherto unclear. Our observation clarifies that any exaggerated decline, though subtle, is likely to reflect a clinical profile characterized by residual symptom burden (i.e., less marked symptom resolution over time). Importantly, this relationship was seen despite our follow-up sample being biased towards better prognostic outcomes (see Supplementary Table 1), as we only scanned patients who were on stable treatment at follow-up. The relationship between lower gyrification and higher illness burden (low n-back accuracy, higher SANS burden and illness duration) was notable even in the larger baseline sample. Thus, the prior results across samples with variable illness durations and symptom states in cross-sectional gyrification studies of schizophrenia may reflect, in part, the presence of patients with more residual symptoms among established cases. Besides, we found the positive and negative symptom-related regions correlated to longitudinal gyrification changes are different, the positive symptoms in the left rostral middle frontal, the negative symptom in the left precuneus and the right rostral middle frontal. In previous studies, the precuneus was widely found associated with negative symptoms^63^, such as apathy^64^. The rostral middle frontal cortex was found related to both negative symptoms^65^ and positive symptoms^66,67^.

Subtle longitudinal changes in LGI after illness onset related to the degree of changes in symptom severity. One possibility is that the trajectory of LGI changes seen after onset reflects the developmental continuum; i.e., those who have a steeper shift in the developmental curve show less treatment responsively. Alternatively, the course of LGI may reflect subtle changes in structural connectivity after illness onset as documented elsewhere^68^; individuals with notable axonal aberrations (reduced integrity) that produce subtle surface-level morphological changes (reduced LGI), may respond less to treatments.

Besides, we did not find a notable association between the course of changes in cortical thickness and gyrification. In our sample, the patients who show the most pronounced reductions in thickness at the outset are not the same ones who show hypogyric patterns, and the regions with a declining pattern of thickness do not overlap with those with a declining pattern of gyrification. In addition, we find no linear correlation between thickness and gyrification in the longitudinal course or in the influence of antipsychotics (supplementary material). In our sample, at baseline, higher LGI in patients was noted in bilateral supramarginal, left superior temporal, and right posterior cingulate, paracentral regions. In contrast, the thickness was mostly reduced at the onset in frontotemporal regions. The frontotemporal and insular thickness further declined in the follow-up sample, though no notable changes in gyrification occurred in these regions. These findings concur with Nelson and colleagues’^24^ observation that over a period of 6 weeks after starting antipsychotics, thickness changes notably, but LGI remains comparatively stable, indicating dissociation. Taken together, these findings do not support the notion of mechanical stress-related progressive reduction; instead, LGI and thickness appear to follow distinct trajectories, carrying specific prognostic information. Overall, our observations negate the mechanical stress hypothesis, but in the context of extant literature, they support the case for a right-shift of the developmental curve for gyrification (See Fig. 3). Accordingly, in schizophrenia, LGI may have an earlier-than-expected peaking and a gradual decrease, but dropping earlier or more quickly during lifespan in patients compared to healthy individuals. This speculation needs to be tested longitudinally with sufficiently long follow-up times.

Our study has many strengths, including a minimally medicated sample, longitudinal follow-up at a clinically stable state, and recruiting a representative sample with sex balance at baseline that captures the population prevalence of schizophrenia^69^. Several limitations should also be taken into account. Firstly, we collected samples at two sites with different acquisition sequences; by treating the scanning site as a covariate in statistical analysis, and ensuring matching controls were recruited at both sites, we adjust for systematic variations, but we cannot quantify the extent to which this might have affected the final results. Secondly, while all patients at baseline were experiencing their first episode, the duration of illness varied greatly; as a result, the baseline sample may have patients at various illness stages, and this may relate to the differences in the time taken for achieving a stable state (3.62 ± 2.58 months). Nevertheless, the first year after the presentation to services is a period characterized by dynamic changes in neurochemistry^70^ as well as brain structure^71^. We report the relationship between DOI and gyrification, but nonlinear effects cannot be ruled out. Thirdly, we did not have a healthy subject sample for longitudinal follow-up. As a result, we refrained from inferring “accelerated LGI decline” in this sample and restricted our interpretations to symptom and treatment correlations to the longitudinal change. Our follow-up sample also differed systematically from those not available for follow-up (attrition bias). Most of these differences come from the fact that the follow-up sample had more female patients; women tend to have later illness onset, somewhat reduced illness duration at presentation and better early outcomes^72^. But the retained group did not differ from the dropped-out group in terms of symptom burden and treatment exposure at onset. We control for sex in all of the reported analyses (also see the supplement for an analysis controlling head size with comparable results). Fourthly, the magnitude of longitudinal changes in gyrification during the relatively short follow-up period in our study is modest, but within the range of lifespan, changes were observed among healthy subjects in larger studies^73,74^. Our careful exclusion of movement-related confounds, the application of longitudinal image registration, and the use of the same scanning parameters over a relatively short time (months) reduced measurement inaccuracy, thus strengthening our interpretation. Finally, we urge readers to practice caution in generalizing our results from symptom-based assessment to real-world functioning; we lacked measures of daily functions for the patients we studied.

Cortical gyrification is aberrantly increased in schizophrenia at presentation, but it is those with lower-than-expected levels of gyrification that display higher symptom burden and reduced treatment response in this illness. Subtle, spatially limited short-term changes in gyrification, but not thickness, occurring in the early phase of treatment related to the inter-individual differences in symptom reduction. By demonstrating the analytical feasibility and retrospective utility of gyrification assessment at first presentation, our work highlights cortical folding as an accessible index to scrutinize prognostic heterogeneity in future studies of psychosis.

## Supplementary information

Supplementary materials

## Data Availability

The data that support the findings of this study are available from the first author, Pan Yunzhi, upon reasonable request.
