# Supplementary material for: Prognostic associations of cortical gyrification in minimally medicated schizophrenia in an early intervention setting
Source: Schizophrenia (Heidelb). 2022 Oct 29;8(1):88. doi: 10.1038/s41537-022-00296-y (PMC9617870; doi:10.1038/s41537-022-00296-y)
Supplement: Supplementary file 1 — Supplementary materials [file 41537_2022_296_MOESM1_ESM.doc]

Supplementary materials list

[Supplementary Table 1 Literature on voxel-wise or vertex-wise comparisons of LGI between schizophrenia and healthy individuals 1](#__RefHeading___Toc114500520)

[Supplementary Table 2 The difference in demography and clinical features between patients with and without follow-up 3](#__RefHeading___Toc114500521)

[Supplementary Figure 1 The relationship between LGI and age at baseline in healthy controls 4](#__RefHeading___Toc114500522)

[Supplementary Figure 2 Correlation between change of negative symptom and LGI at baseline 5](#__RefHeading___Toc114500523)

[Supplementary Figure 3 No correlation between voxel-wised gyrification and dosage of antipsychotic medication at baseline 6](#__RefHeading___Toc114500524)

[Supplementary Figure 4 Higher daily and total dosage of antipsychotic medication during the follow-up period indicate focal reduction in LGI and cortical thickness 6](#__RefHeading___Toc114500525)

[Supplementary Figure 5 (SF5): Violin plots displaying the spread of continuous variables in Table.1 8](#__RefHeading___Toc114500526)

[Supplementary Materials Results with eTIV as one of covariates 8](#__RefHeading___Toc114500527)

# Supplementary Table 1 Literature on voxel-wise or vertex-wise comparisons of LGI between schizophrenia and healthy individuals

To provide a synthesis of prior evidence from voxel-wised comparison of LGI between schizophrenia and healthy controls, we undertook a systematic literature search. The databases MEDLINE, BIOSIS Previews and Web of Science were searched, from time to conception to 1st December 2020, for any original journal articles, written in English. The search strategy combined abstracts and keywords and was primarily applied trough the portals of Web of science and Pubmed and adapted for the other databases. We used the keywords “schizophrenia”, “gyrification OR cortical folding” and “MRI”. After removing duplicates, the list of initial articles was processed through the inclusion and exclusion criteria given below.

Inclusion criteria were as follows: 1) an original, peer reviewed journal article, 2) studying human participants, 3) investigated gyrification using voxel-wised method, 4) recruited patients with schizophrenia confirmed through a diagnostic interview and compared them against healthy volunteers, 5) in English language and 6) a case-control or longitudinal study. Additional inclusion criteria for the accompanying meta-analysis were a) the reporting of standard space coordinates for the maxima of significant voxels/clusters and b) employing a whole-brain between-group analysis. Studies that did not meet the above criteria or met the following exclusion criteria were not included in the review: 1) any subjects in the patient sample with a principal psychiatric diagnosis other than schizophrenia (e.g. bipolar disorder, MDD, etc.), 2) any subjects in the schizophrenia group with a concurrent neurological condition, 3) studies that fail to report on a measure of gyrification (e.g. reporting only gray matter volume) only 4) the study design was a case report, or a case-series. Where authors met these criteria but had not reported stereotactic coordinates, they were contacted and asked to provide this information.

Finally we found 19 studies met our criteria. And 16 of 19 studies have more than 3 years exposure to antipsychotics, as discussed in introduction.

| **Supplementary Table.1**  **The literatures about voxel-wised comparison of LGI between schizophrenia and health controls** | | | | | | | | | |
| --- | --- | --- | --- | --- | --- | --- | --- | --- | --- |
| ID | Authors, Year | Sample sizes (F/M) | | Age in years±SD | | Medication | Mean positive syndrome score | Mean negative syndrome score | Cross-sectional whole brain voxel-wised results (SCH vs. HC) |
|  |  | SCH | HC | SCH | HC |  |  |  |  |
| 1 | Haukvik et al. 2012 | 54 (17/37) | 54 (21/33) | 41.9±8.0 | 41.5±8.9 | All medicated | 3.9±2.72 | 7.6±5.1 | Uncorrected results (at p<0.05) demonstrated lower gyrification in patients than in healthy controls in several regions across both hemispheres |
| 2 | Palaniyappan et al. 2012 | 57 (7/50) | 41 (2/39) | 26.1±7.5 | 28.0±6.6 | All medicated | NA | NA | Whole-brain analysis revealed 4 clusters in the left hemisphere and a single cluster in the right hemisphere with significant reduction in gyrification in patients compared with controls. |
| 3 | Palaniyappan, et al. 2013 | 18 (8/10) | 19 (10/9) | 16.1±1.15 | 15.7±1.27 | All medicated | PANSS positive score 22.67(3.16) | PANSS negative score 15.67(2.92) | Baseline hypergyria was noted in a cluster that included Broca's area (pars opercularis/pars triangularis of left inferior frontal gyrus [IFG]) and adjacent left insula, but this progressively reduced. Significant hypogyria was noted in the right superior temporal cluster. |
| 4 | Palaniyappan et al. 2013 | Psychosis 80 (22/58) | 46 (21/25) | Psychosis 28 ±8.2 | 24.7 ±5.6 | Including 12 Treatment-naive subjects | PANSS positive score 15.5(5.0) | PANSS negative score 15.9(5.5) | Patients had significant reductions in gyrification in the middle/inferior frontal gyrus, precentral gyrus, and precuneus in the left hemisphere and the middle frontal gyrus and inferior parietal (angular gyrus) region in the right hemisphere. |
| 5 | Palaniyappan et al. 2014 | 39 (7/50) | 34(2/39) | NA | NA | Almost All medicated | NA | NA | Significant group differences were also noted in gyrification, with more prominent reduction in the SCZP. |
| 6 | Nesvåg et al. 2014 | Sample 1  95(25/70)  Sample 2  112(46/56) | Sample 1 106 (34/72) Sample 2 100 (58/42) | Smaple 1 42.2±7.1 Sample 2 31.8±8.6 | Smaple 1 41.5±9.0 Sample 2 37.6±10.2 | All medicated | Sample 1  18.6 (26.5) Sample 2  NA | Sample 1 28.0 (19.0) Sample 2  NA | In the combined sample, patients had significantly lower lGI in three clusters; left lateral pericentral cortex, right temporo-occipital cortex, and right medial parietal cortex. Similar clusters were found when the Swedish and Norwegian samples were analyzed separately. |
| 7 | Schultz et al. 2014 | 63 (NA) | 65 (NA) | 26.15±7.45 | 28.15±10 | All medicated | PANSS positive, mean (S.D.) 17.7 (7.8) | PANSS negative, mean (S.D.) 17.75 (8.55) | No significant cortical folding differences between patients and controls. |
| 8 | Besteher et al. 2016 | 37 (21/16) | 50 (25/25) | 31.6±8.8 | 29.5±7.9 | All medicated | PANSS pos  16.9 (10.3) | PANSS neg. 19.6(7.9) | Significantly increased gyrification (p=0.001) in patients in the right medial temporal cortex |
| 9 | Sasabayashi et al. 2017 | 57 (33/24) | 62 (36/26) | 25.1±4.7 | 24.1±4.7 | All medicated | 27.8 (22.0) | 53.2 (25.6) | Patients with first-episode schizophrenia had a significantly higher LGI in the bilateral superior frontal gyri, bilateral frontal poles, bilateral medial and lateral orbitofrontal cortices, bilateral rostral anterior cingulate gyri (ACG), left paracentral lobule, right rostral middle frontal gyri, right caudal anterior and posterior cingulate gyri, right lateral parietal cortices, and bilateral occipital regions |
| 10 | Sasabayashi et al.2019 | 101(55/46) | 77 (44/33) | 25.6±5.5 | 24.2±5.7 | All medicated | 27.9 (21.4) | 49.1 (21.1) | Patient groups had a significantly higher LGI in cortical regions, including bilateral prefrontal and left parietal cortices, than controls. |
| 11 | Spalthoff et al. 2018 | 51 (17/34) | 102 (33/69) | 35.18±10.88 | 33.15±9.6 | 7 without antipsychotic medication.43 received | 19.38 (11.75) | 42.45 (15.30) | Schizophrenia patients had a significantly higher LGI in broad regions than healthy controls, predominantly in the bilateral medial frontal regions. |
| 12 | .Kubera et al. 2018 | PAVH  10(4/6)  nPAVH  10(2/8) | 14 (7/7) | PAVH  36.5±9.0 nPAVH  32.1±6.2 | 33.7±8.6 | All medicated | PANSS-P PAVH 16.0(3.4) nPAVH 11.2(3.2) | PANSS-N PAVH 22.0(5.5) nPAVH 18.8(5.7) | Patients with auditory verbal hallucinations (PAVH) showed lower LGI in Broca's region compared to both healthy controls and nAVH patients |
| 13 | Takayanagi et al.2018 | 73(40/33) | 50(25/25) | 26.9±6.55 | 25.8±4.70 | All medicated | 9.25(7.8) | 14.45(9.5) | Patients exhibited hyper-gyric patterns in the bilateral dorsal medial prefrontal and ventromedial prefrontal cortices, bilateral anterior cingulate gyri and right lateral parietal/ occipital cortices as compared with HCs. |
| 14 | Nelson et al. 2019 | 34(25/9) | 23 (19/4) | 28.3±9.42 | 27.5±9.63 | 12 subjects without antipsychotic medication.22 received | BPRS-P  10.44 (3.47) | BPRS-N  7.44 (3.16) | HC showed greater baseline LGI compared to SZ bilaterally throughout the fronto-temporal areas, including the insula and pre/postcentral gyri, as well as the left temporo-parietal junction and right supramarginal gyrus. |
| 15 | Madre et al. 2019 | 128(74/54) | 127(73/54) | 41±10 | 39±10 | All medicated | PANSS-P 13±5 | PANSS-N 17±6 | Patients had reductions in gyrification of temporal poles, superior temporal gyrus, insula, parietal areas (mainly supramarginal and postcentral gyri) and frontal caudal regions. |
| 16 | Madeira et al. 2020 | 20(7/13) | 20(7/13) | 31.5±10.3 | 31.5±10.3 | All medicated | NA | NA | In right inferior frontal gyrus – BA47, the SCZ group had lower gyrification compared to healthy controls. |
| 17 | Zhou et al. 2021 | 41(23/18) | 30(17/13) | 28.83±10.22 | 27.47±7.89 | Drug-naive | PANSS-P 24.88±6.75 | PANSS-N 21.80±9.15 | Patients had higher cortical gyrification in the left lateral occipital cortex, but lower cortical gyrification in the left transverse temporal cortex. |
| 18 | Pham et al. 2021 | 23(8/15) | 39(17/22) | 23.5±4.8 | 24.6±4.7 | All medicated | SAPS 29.0±24.3 | SANS 52.1±25.5 | The schizophrenia group exhibited a progressive decline in LGI, predominantly in the fronto-temporal regions, whereas LGI increased over time in several brain regions in the schizotypal and control groups. |
| 19 | Rosa et al. 2021 | 240 (84/156) | 243 (94/149) | 34.87±9.82 | 33.24±9.94 | All medicated | PANSS-P 13.16±4.68 | PANSS-N 17.48±5.67 | The chronic schizophrenia and HC groups did not differ in terms of cortical thickness, area or LGI in the whole-cortex comparisons. |

# Supplementary Table 2 The difference in demography and clinical features between patients with and without follow-up

We undertook a comparison between patients with and without longitudinal follow-up. The results are as below. All patients were on stable medication and did not receive any specific therapy other than supportive psychotherapy offered during clinical follow-ups. Patients who were followed up were mostly female, older in terms of their current and onset age, and had shorter duration of illness. This profile and accompanying attrition bias may imply a tendency for patients with better outcome profile to be retained for follow-up, indicating that the small but progressive hypogyria may be even more evident in more unbiased samples.

| Supplementary Table 2: The difference in demography and clinical features between patients with and without follow-up | | | |
| --- | --- | --- | --- |
|  | Patients with follow-up (n=40) | Patients without follow-up(n=68) | P value |
| Center (CS/HK) | 18/22 | 68/0 | **<0.001** |
| Sex (M/F) | 15/25 | 47/21 | **0.001** |
| Age (year) | 27.28(10.51) | 23.62(5.49) | **0.046** |
| Onset age | 26.75(10.75) | 22.12(5.30) | **0.014** |
| Duration of illness (month) | 7.90(10.79) | 19.35(26.58) | **0.002** |
| Daily dosage of Medication (CPZ/day) | 205.07(187.67) | 228.39(190.78) | 0.539 |
| Total dosage of Medication (CPZ) | 1409(1929) | 1594(1900) | 0.628 |
| Medication time (day) | 6.10(4.71) | 6.50(4.25) | 0.651 |
| Total score of symptom | 48.63(25.07) | 53.90(34.60) | 0.402 |
| SAPS score | 23.73(13.50) | 23.16(15.58) | 0.849 |
| SANS score | 24.90(21.86) | 30.74(26.12) | 0.237 |
| N-back (accuracy rate) | 0.65(0.18) | 0.53(0.22) | 0.057 |
| Estimated total intracranial volume | 1.46E+6 (2.07E+5) | 1.48E+6(2.56E+5) | 0.697 |
| Note: CS, Changsha city; HK, Hongkong City; M, male; F, female; CPZ, chlorpromazine. | | | |

# Supplementary Figure 1 The relationship between LGI and age at baseline in healthy controls

Figure below showed the correlation analysis between LGI and age at baseline **in healthy controls**. A general linear model using QDEC with center and sex as covariates, with multiple testing correction performed using permutations through Monte-Carlo simulations (n = 10000) and identified clusters that survived a type-1 error rate of 5% at a cluster inclusion threshold of P = 0.01.The results indicated lower prefrontal LGI in older healthy controls, as expected (See also Figure 4 for age-LGI relationship model).

| Supplementary Table 4: LGI an age relationship among healthy subjects | | | | |
| --- | --- | --- | --- | --- |
| Cortical regions | The direction of correlation | Peak vertex coordinate | Cluster size (mm^2^) | Cluster-wise  probability |
|  |  | x, y, z |  |  |
| Left superior frontal | Negative | -12.8, -11.1, 67.7 | 2339.59 | 0.00010 |
| Right superior frontal | Negative | 13.7, -2.3, 62.9 | 850.00 | 0.00010 |


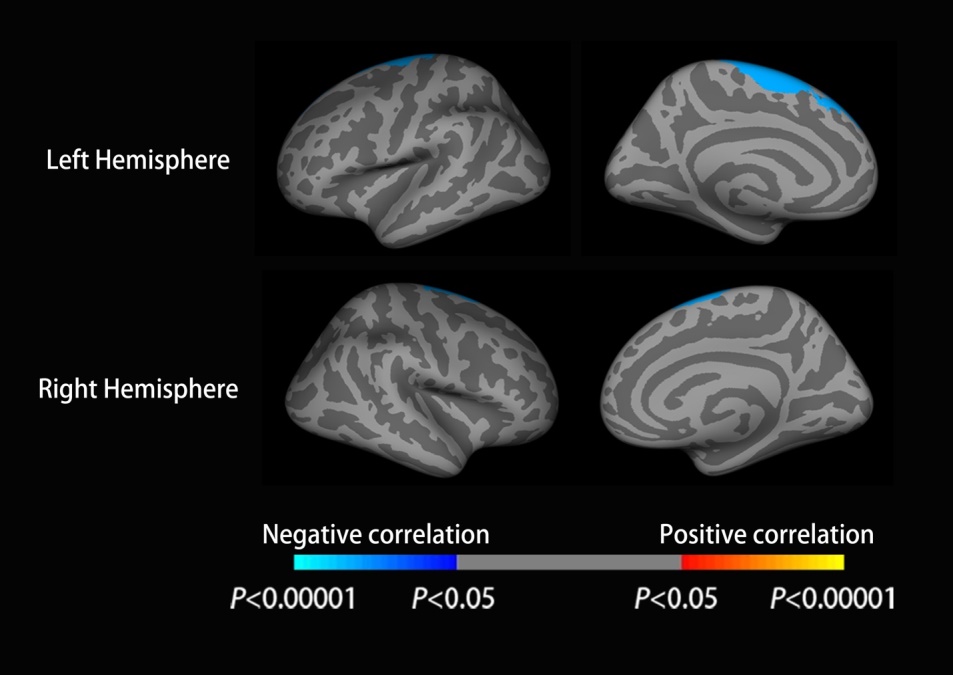
Supplementary Figure 1 (SF1): The relationship between LGI and age at baseline in healthy controls

# Supplementary Figure 2 Correlation between change of negative symptom and LGI at baseline

This figure SF2 displays the correlation between the change in negative symptom severity over time with the baseline LGI across the cortex. A general linear model controlling for the effect of age, center, sex and follow up time was used to compute the correlation between LGI at baseline and the change [(Baseline – follow up)/Baseline] of negative symptom at each vertex of the right and left hemispheric surfaces. To correct for multiple testing, we used Monte-Carlo simulations (n = 10 000) and identified clusters that survived a type-1 error rate of 5% at a cluster inclusion threshold of P = 0.01. Cortical mapping showed that LGI in bilateral superior temporal and left pericalcarine at baseline was negatively correlated decrease rate of SANS scores.


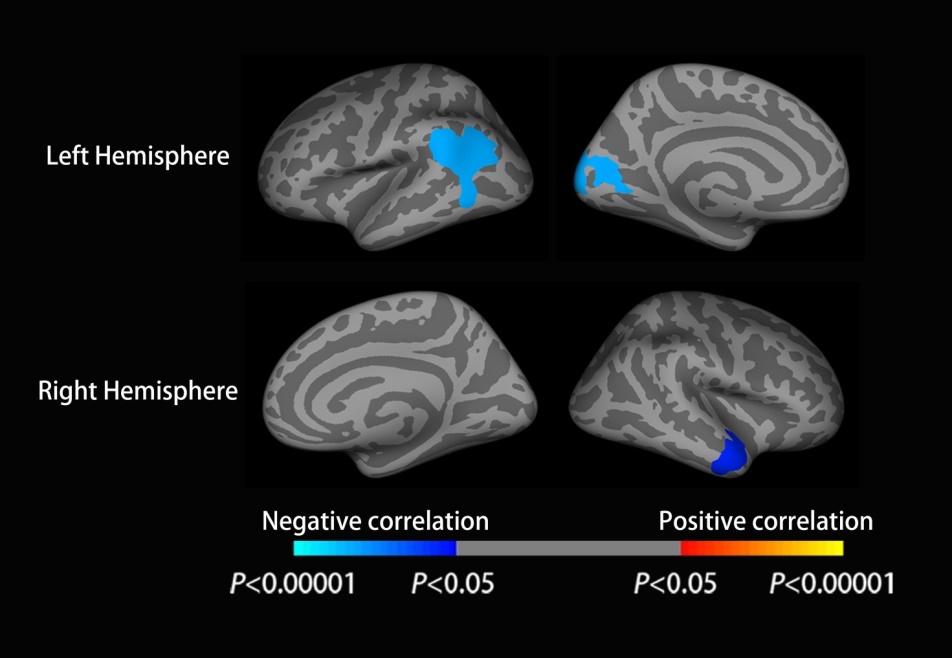


Supplementary Figure 2 (SF 2): Correlation between change of negative symptom and LGI at baseline

# Supplementary Figure 3 No correlation between voxel-wised gyrification and dosage of antipsychotic medication at baseline

Cortical statistical maps below (SF3) display the correlation between defined daily doses and the LGI or CT at baseline in patient group. The results showed no correlation between gyrification and dosage of antipsychotic medication at baseline. But we found patients with lower cortical thickness in superior parietal region (at baseline) were more likely to be receiving higher doses of antipsychotics at baseline. Interestingly, we also found a negative correlation (Pearson r=-0.216, p=0.024) between cortical thickness in the same superior parietal region and visual hallucination severity (SAPS Item 6), suggesting that a higher symptom burden (hallucinations) in those with thinner superior parietal cortex might have influenced the higher antipsychotic doses at baseline. Direction of these relationships cannot be disentangled using the cross-sectional baseline data.


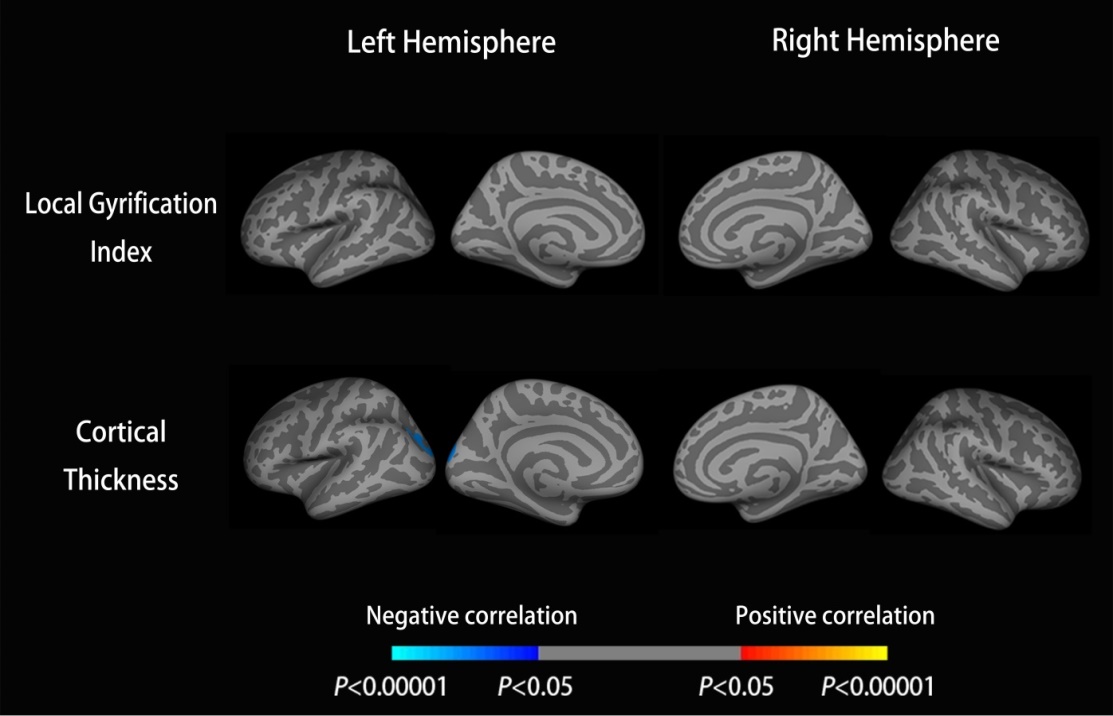


Supplementary Figure 3 (SF3): The relationship between gyrification and dosage of antipsychotic medication at baseline

# Supplementary Figure 4 Higher daily and total dosage of antipsychotic medication during the follow-up period indicate focal reduction in LGI and cortical thickness

In the smaller follow-up sample, we found a linear correlation between longitudinal changes (Baseline – Follow up) in cortical measures (thickness/ gyrification) and dosage of antipsychotic medication during the follow-up period. The higher the dose of antipsychotic medication during the follow-up, the higher the magnitude of reduction in LGI and cortical thickness. But these changes were spatially circumscribed and survived only a lenient threshold of p=0.01.


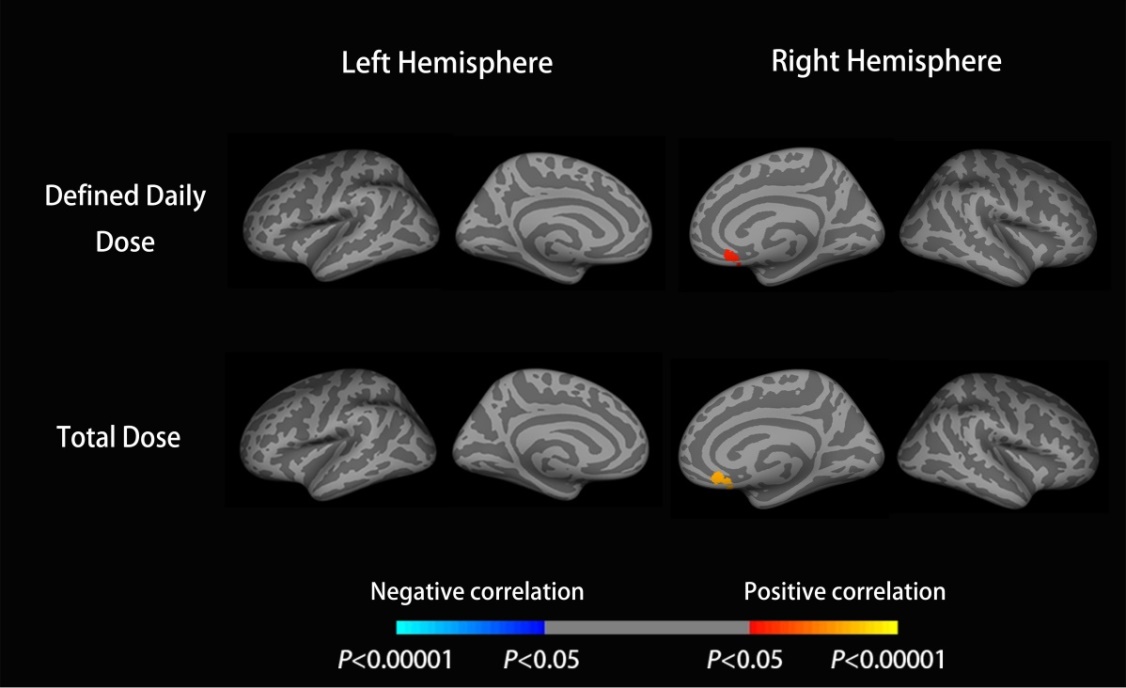


Supplementary Figure 4A (SF4A). Cortical statistical maps displaying the correlation between **the** longitudinal **change in LGI** and Defined Daily Dose and total dose in the patient group during the follow up phase. Higher dose exposures relate to more LGI reduction over time in circumscribed orbitofrontal region at a lenient cluster inclusion threshold p=0.01.


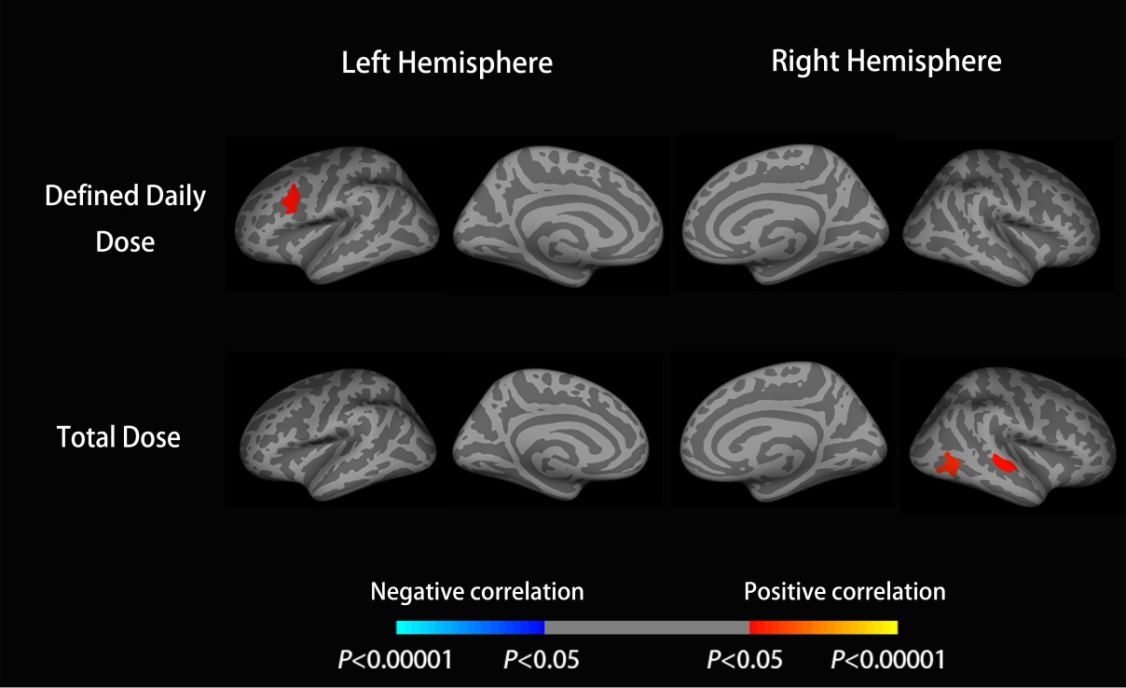


Supplementary Figure 4B (SF4B). Cortical statistical maps displaying the correlation between **the changes in thickness** and Defined Daily Dose and total dose in the patient group during the follow up phase. Higher dose exposures relate to more thickness reduction over time in circumscribed frontotemporal regions at a lenient cluster inclusion threshold p=0.01.

# Supplementary Figure 5 (SF5): Violin plots displaying the spread of continuous variables in Table.1


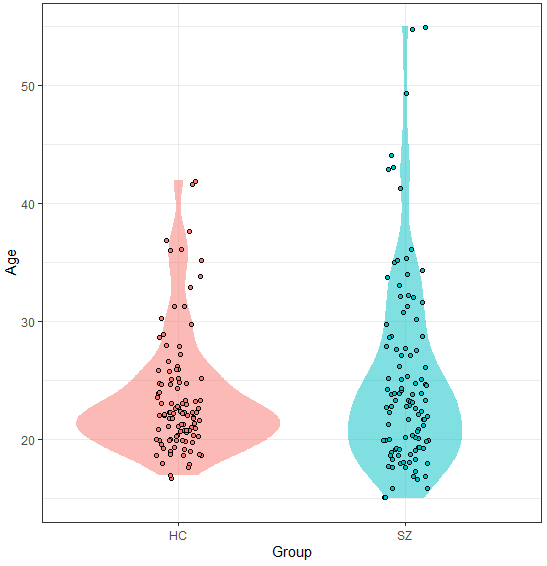

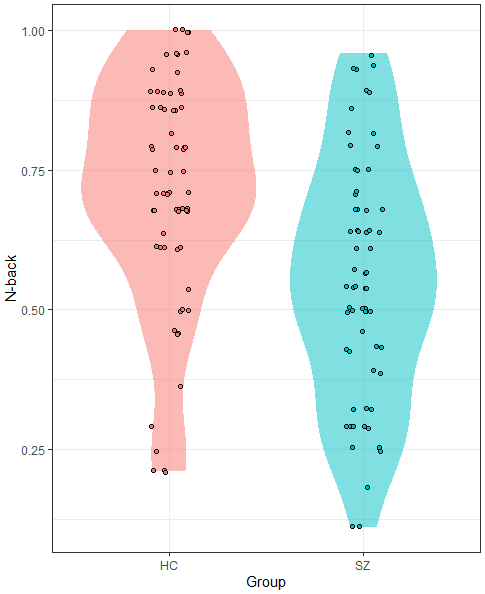

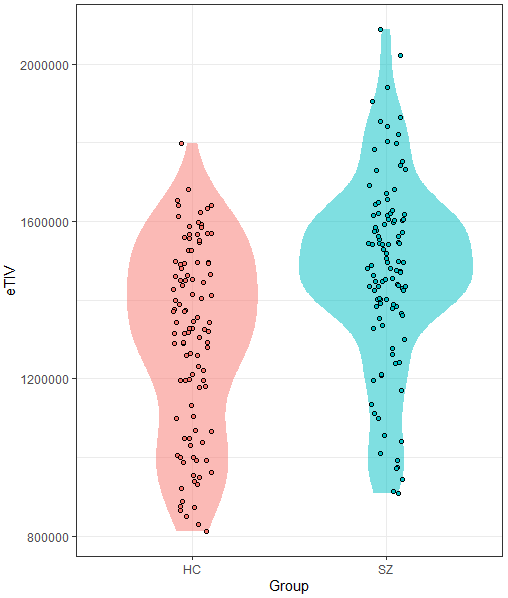


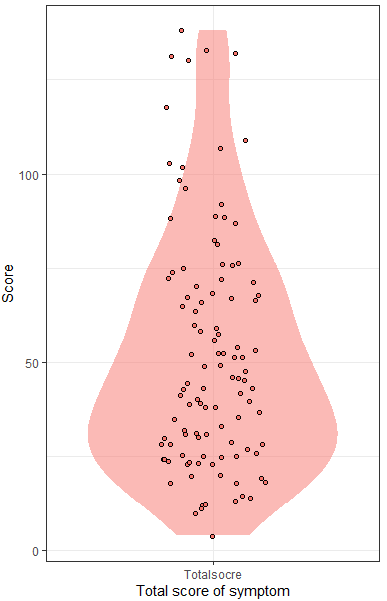

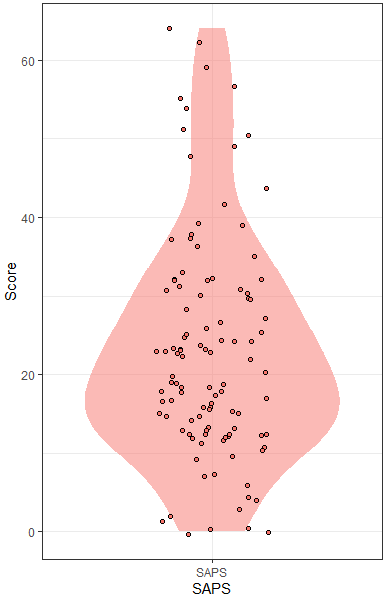

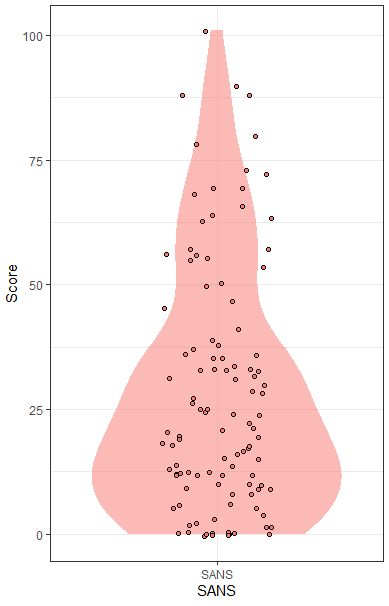

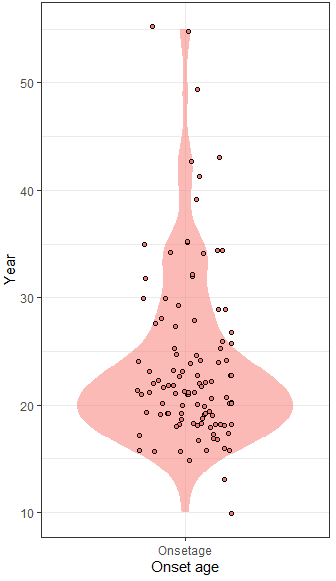

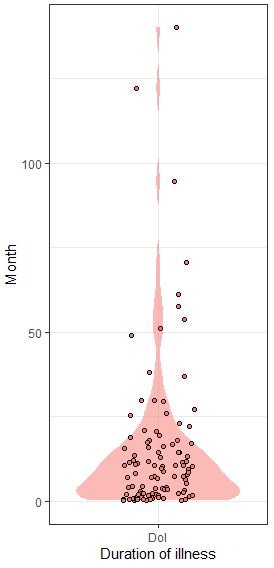

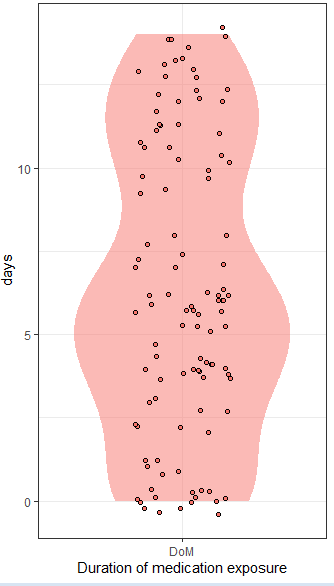

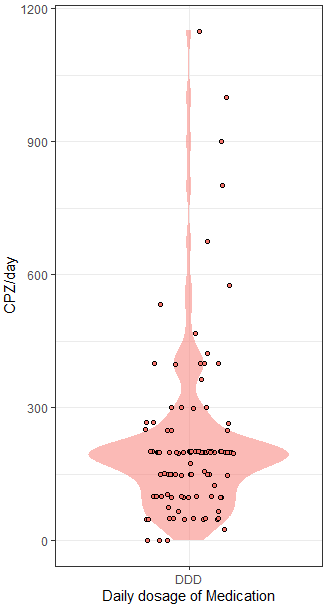


Supplementary Figure 5 (SF5): Violin plots displaying the spread of continuous variables in Table.1

# Supplementary Materials Results with eTIV as one of covariates

As our sample had higher estimated intracranial volume in patients compared to controls at baseline, here we present the results of all major analyses after adjusting for **eTIV as a covariate**.

3.1. Baseline group difference in gyrification

Whole-brain vertex-wised group analysis with center, sex, age and **eTIV as covariates** revealed a single cluster in the left hemisphere and 3 clusters in the right hemisphere with the significantly higher LGI in patients compared to healthy controls. These clusters included bilateral supramarginal, right cingulate and fusiform (between the lateral occipital and inferior temporal regions). In a subsequent analysis, significantly lower cortical thickness was observed in right pars opercularis and middle temporal regions in patients compared to healthy controls. The largest cluster centered on pars opercularis. Meanwhile, we observed higher cortical thickness in left caudal middle frontal and superior parietal in patients compared with healthy controls. The details of group differences are shown in SF7 and Supplementary Table 5 below.

| Supplementary Table 5: Baseline group difference in gyrification after adjusting for eTIV | | | | |
| --- | --- | --- | --- | --- |
| Cortical regions | Change direction in patients | Peak vertex coordinate | Cluster size (mm^2^) | Cluster-wise  probability |
|  |  | x, y, z |  |  |
| Local gyrification index |  |  |  |  |
| Left supramarginal | Higher | -57.0, -34.2, 40.4 | 782.82 | 0.00810 |
| Right supramarginal | Higher | 52.9, -44.2, 28.9 | 969.86 | 0.00030 |
| Right caudal anterior cingulate | Higher | 7.7, 11.7, 27.1 | 828.42 | 0.00190 |
| Right fusiform | Higher | 34.3, -73.5, -12.0 | 706.79 | 0.00820 |
| Cortical thickness |  |  |  |  |
| Left caudal middle frontal | Higher | -29.1, 0.6, 48.6 | 196.68 | 0.00030 |
| Left Superior parietal | Higher | -23.9, -78.5, 16.8 | 149.50 | 0.00420 |
| Right parsopercularis | Lower | 41.3, 13.9, 21.0 | 305.24 | 0.00010 |
| Right middle temporal | Lower | 59.4, -45.3, -6.9 | 157.63 | 0.00220 |


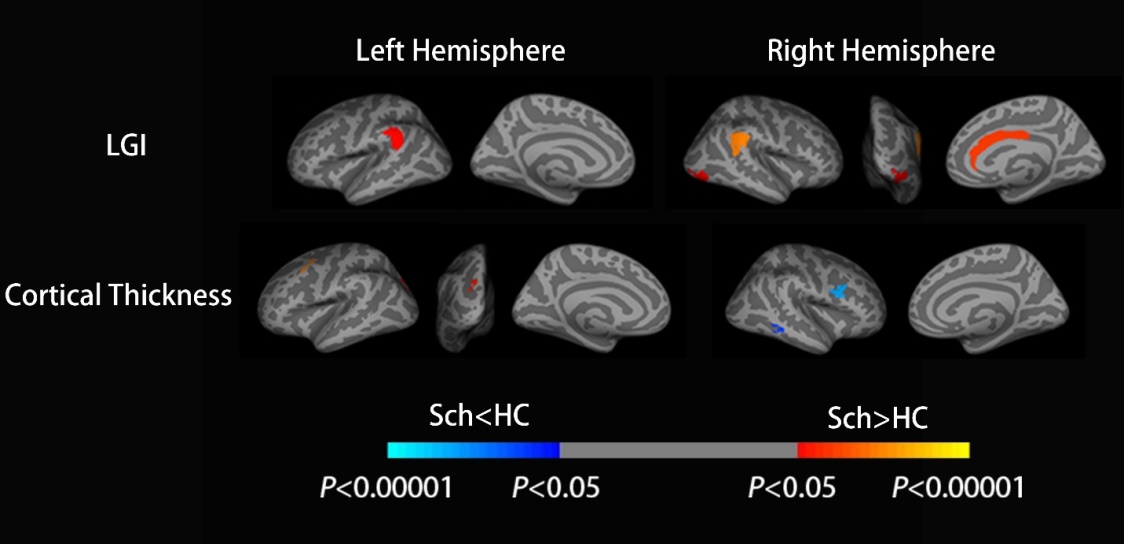


Supplementary Figure 7 (SF7): Clusters showing differences in the LGI, cortical thickness between schizophrenia patients and healthy controls with center, sex, age and **eTIV as covariates**. The statistical maps display higher LGI in patients compared to healthy controls. The maps are shown for the bilateral supramarginal, right cingulate and fusiform, respectively. The horizontal bar shows p values corrected for multiple comparisons.

3.2. Correlational analysis at baseline

At baseline, we analyzed the correlation between LGI with duration of illness (DoI), SAPS total score, SANS total score, and n-back performance in drug-naive patients (shown in Figure B). A negative correlation was observed in the bilateral precuneus and left insula between LGI and DoI (longer DoI seen in those with lower LGI), and between caudal anterior cingulate and SANS total score (higher negative symptom burden in the presence of lower LGI), but no corrected cluster correlated with SAPS. Lower N-back accuracy was associated with lower LGI in left rostral middle frontal and right superior parietal regions. Taken together, lower LGI at various regions at the outset (baseline) related to many indicators of more severe illness.


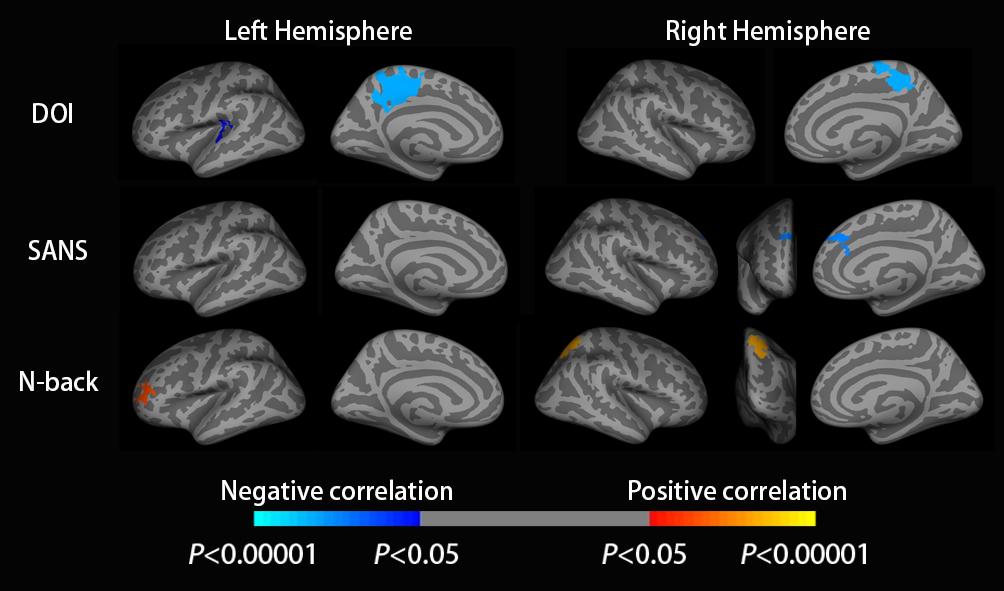


Supplementary Figure 8 (SF8): Cortical statistical maps displaying the correlation between the LGI and clinical variables. The horizontal bar shows p values corrected for multiple comparisons.

3.3. The relationship between longitudinal changes of gyrification and clinical changes

In the longitudinal analysis, the changes of gyrification were calculated using [Follow up - Baseline] / Baseline. Longitudinally, reductions of gyrification were observed in left postcentral (CWP=0.007) and right precentral (CWP=0.0077), increase of gyrification was observed in right rostral middle frontal (CWP=0.0001) (Figure C). After covariate for follow up time, imaging center, sex, age at baseline and eTIV, better treatment efficacy (greater reduction in total SAPS plus SANS scores) was found in patients who experienced smaller reductions in gyrification compared to baseline in left inferior parietal (CWP=0.0001), left supramarginal(CWP=0.0001), left superior parietal (CWP=0.0001), right precentral (CWP=0.0001), and greater reductions in gyrification in the left medial orbitofrontal cortex (CWP=0.0001) compared to baseline.

When the analysis was limited to improvements in positive symptoms only (decrease in total SAPS score), greater symptom reduction related to smaller reductions in gyrification from baseline in left rostral middle frontal, left precentral, left superior temporal, left inferior parietal, but greater reductions in gyrification compared to baseline in the right superior parietal cortex. When limited to improvements in negative symptom, greater reduction in total SANS score was related to smaller reductions in gyrification of left precuneus, left supramarginal gyrus, left inferior parietal lobule, right rostral middle frontal gyrus, right postcentral gyrus, but greater reductions in gyrification compared to baseline in left rostral middle frontal gyrus.


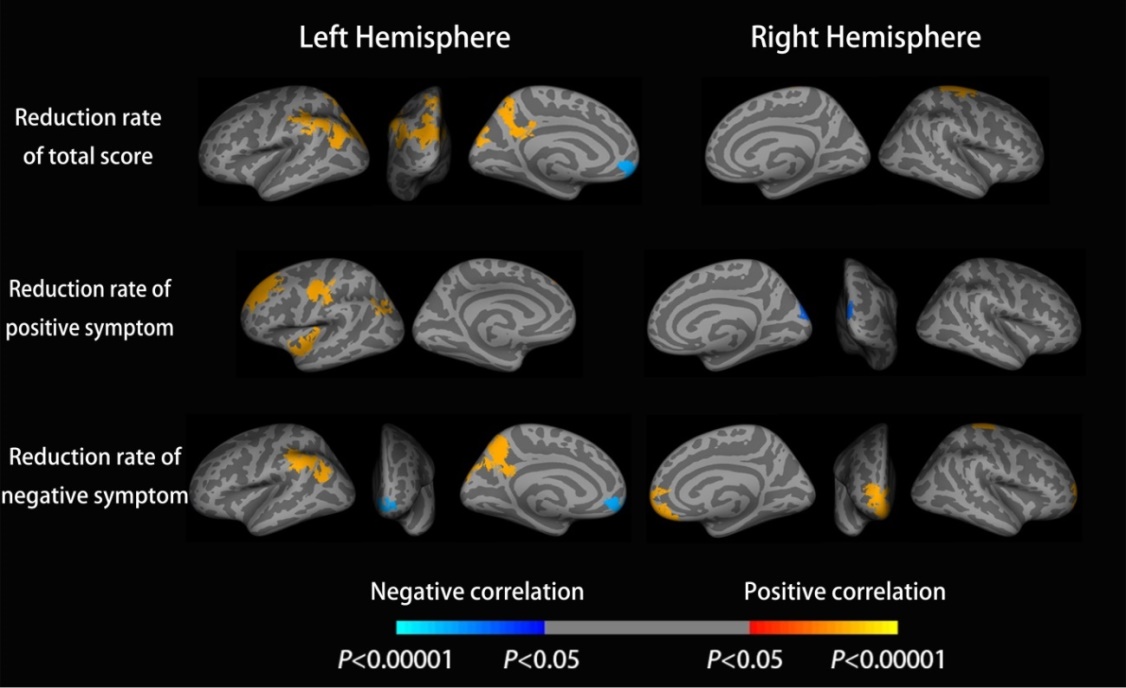


Figure C. Cortical statistical maps displaying the correlation between the longitudinal gyrification changes and changes in symptom burden . The horizontal bar shows p values corrected for multiple comparisons (Monte-Carlo permutations). Gyrification changes = [Follow up LGI – Baseline LGI] / Baseline LGI. Clinical changes = [Baseline scores – Follow-up scores]/ Baseline scores. Positive and negative symptom scores based on summary scores of corresponding PANSS items.
